# Pandemic, Quarantine, and Psychological Time

**DOI:** 10.3389/fpsyg.2020.581036

**Published:** 2020-10-20

**Authors:** Simon Grondin, Esteban Mendoza-Duran, Pier-Alexandre Rioux

**Affiliations:** École de Psychologie, Université Laval, Québec, QC, Canada

**Keywords:** quarantine, perception of time, pandemic (COVID-19), psychological time, time judgments

## Abstract

This article addresses the feeling of strangeness about the perception of time that many people with ordinary lifestyles experienced during the quarantine imposed to fight the presence of COVID-19. It describes different aspects of psychological time affected by the interruption of a normal routine and suggests some cognitive mechanisms, attention, and memory that might have been at play, leading to perceive time as being more or less long. The article also describes the critical role of anxiety and temporal uncertainty and how they may affect the functioning of an internal clock and reminds the reader that there are individual differences in time-related aspects of the personality that contribute to the variety of impressions about duration experienced during the quarantine.

## Introduction

The pandemic caused by COVID-19 led many governments, early in 2020, to impose a mandatory quarantine to their population. All of a sudden, many people were required to stay at home. The severity of the quarantine varied across countries, with some people having to restrict their trips to groceries, while other people below the age of 70 years old were allowed to walk around, as long as they stayed 2 m away from one another. Conditions at home varied considerably, first by the need to keep working from home or not, and by the space conditions of daily life, which include the fact of sharing this space with others, sometimes with young children. During this quarantine, most people had, at some point, the impression of being disorganized, and at the heart of this impression was a complaint about time. The World Health Organization (WHO) has reported that the current situation has affected normal activities, routines, and schedules in most parts of the world, and most people were touched by the situation. Having this into consideration, the WHO recommended people to try to stay as organized as possible, respecting routines, or even creating new ones even if sometimes it is not an easy task (World Health Organization, 2020). This time-related feeling of strangeness may have to do with the fact of having to stay at home. For many people, staying at home can generate boredom. This boredom can affect the relation individuals have with time, time appearing sometimes as extremely long and monotonous (Kumar and Nayar, 2020).

In this paper, we will try to capture what aspects of psychological time were disturbed by the quarantine imposed by the pandemic and what was at play from a temporal viewpoint. Even if there are other aspects, like the amount of space available during the lockdown that may affect personal mood and perception, the purpose of the present paper is to analyze the impact on time perception of changes, like the adoption of new activities or new schedules, provoked by the quarantine. The impact of these changes will be partly analyzed in the light of an internal-clock perspective (Block and Zakay, 1997).

## Psychological Times

There are dozens of research avenues in the field of time perception and psychological time (see Grondin, 2020). Before pointing out what went wrong, it is good to remind that, in the universe of timing and psychological time, many things were not changed. Adult people did not regress in many time-related aspects of adaptation. They were still able not to confuse, like very young children do, the interactions between speed, space, and time (Piaget, 1946). Adults remained able to use past, present, and future tenses correctly when using language, which is also demanding for very young children (Wagner, 2001). Musicians kept being able to play with the correct rhythm and tempo. There is also no reason to believe that the knowledge of chronometric units (e.g., minutes, days, and months) did not remain intact, although we know that a complete 2-week confinement (with no cues to real time of day) will lead to a plus or minus 3 h accuracy in the estimation of the time of the day (Thor and Crawford, 1964).

To capture what may have gone wrong, it is useful to remind that there are paradoxical impressions about time that were already there before quarantine. You can ask an elderly person about the impression of the speed of time right now, and if this person has no visitors or no activities on their schedule, in brief, if it is a boring period, the answer would likely be that time passes very slowly. However, if you ask the same person a minute later how fast the past 10 years passed, or the past 20 years, it is likely that this person will say that time has passed very quickly (“I haven’t seen the time go by”).

This paradox could be at least partially explained by a crucial distinction about the mechanisms of time perception and psychological time that are kept in mind. One distinction is about the fact that judgments about the passage of time are not duration judgments (Droit-Volet and Wearden, 2015, 2016; Wearden, 2015). In the former case, one refers to judgments about how fast time seems to pass in some situations. What is referred to as duration judgments is the type of investigation based on an explicit judgment about the duration of an interval, or the duration of an activity or task. Both types of judgments are not related, according to Droit-Volet and Wearden (2016). The judgments about the passage of time are indeed closer to phenomenology and, as noted by Droit-Volet and Wearden (2016), these judgments are linked to the individuals’ introspection on their internal life. The paradox described in the previous paragraph may be seen as reflecting the variety of temporal avenues belonging to the internal life.^1^

The second distinction is actually related specifically to duration judgments: prospective vs. retrospective timing (Brown, 1985). In retrospective timing, a person does not know in advance that a temporal estimation of a given temporal extent (duration of a task or activity) will have to be made; in prospective timing, the individual is informed beforehand by an experimenter that time must be estimated. Retrospective timing is reported to rely heavily on memory mechanisms (Block and Zakay, 1997). But when you know that you have to estimate time (prospective timing), the perceived duration of an interval will depend heavily on the attention allocated to time, with more attention to time resulting in a longer perceived duration. Such a finding is very commonly reported when intervals to be judged are relatively brief, from 100 ms to several seconds (Macar et al., 1994; Block and Zakay, 1997), but also applies to much longer durations, up to 42 min (Bisson and Grondin, 2013) and 58 min (Tobin et al., 2010). The type of paradox described two paragraphs ago could be caused by the involvement of different timing mechanisms: without activities at a given moment, more attention is susceptible to be allocated to time, which leads to the impression that time is long, while the impression about a long time period in the past must rely on memory mechanisms.

This effect of attention on time perception is often embedded within one classical viewpoint on time perception: there is an internal clock, a clock described as a pacemaker-counter device. The pacemaker emits pulses that are accumulated by the counter, with a higher accumulation resulting in longer perceived duration. Using this model is useful because it helps to account for both the effect of attention and the effect of internal physiological changes (arousal) on duration judgments. This pacemaker-counter process is indeed under the control of attentional processes. For instance, in double task situations involving a temporal task and a nontemporal task, when more attention is allocated to the nontemporal task, less attention is directed to time, which leads to a smaller accumulation of pulses and results in shorter perceived duration (Block and Zakay, 1997; Grondin, 2010). In the quarantine situation caused by COVID-19, different aspects may disturb attention and attention to time: for instance, having to stay at home, adopting new routine or new habits, and waiting for news from employers or schoolboards. In this context, it is crucial to keep interest in activities, would it be TV series, films, reading, listening to music, playing cards, doing puzzles, etc., in order to make sure attention would not be directed toward time. From an attentional viewpoint, in the context of quarantine, the act of focusing on time or on something else likely contributed to the impression that some time intervals seem brief or long.

Several aspects of psychological time are located in memory processes, as is the case for retrospective timing for instance (see Block and Zakay, 2008). But relying on memory mechanisms to estimate time comes with some failures and biases. We rely on memory not only to estimate the duration of past activities but also to locate an event in the past and to compare the time elapsed since certain events (Friedman, 1993). In the specific case of the estimation of event duration, it is known that judgments are based on the knowledge of the duration of similar events (Burt, 1993). In order to estimate the duration of an event, humans rely on their memory, comparing the duration of the current event with the duration of similar events. The difficulty to estimate the duration of some events during the lockdown might come from the fact that people are experiencing a situation for the first time.

## Losing the Notion of Time

Quarantine leads to the interruption of normal activities. What was interrupted is indeed a series of landmarks in the regular citizen’s schedule. In addition to losing a schedule at work to remind us which day we are, there were no more outings to the theater on Thursday or to the cinema on Friday, or dinner with friends or family on Saturday or Sunday, or services at the church, or a favorite program or professional sports game on TV on a week day. Slowly, the days have lost their identities, their specific flavor, and the name of the days has lost some of its significance. With the loss of these landmarks in the week, or in the month, people were exposed to losing their motivation (Dai and Li, 2019).

At the same time, new habits were taken, with little association with specific days of the week. There was a serious increase of the time spent on screens, especially for children (Wiederhold, 2020). This situation makes average citizens who have experienced the loss of their daily temporal landmarks realizing the extent to which our lives were previously scheduled, conditioned. Instead, with the disruption of time that people might have experienced, time sense was recovering its right to adjust to a more natural pace; in modern occidental life, there is a need to synchronize activities, to have frequent precise timing of events relative to other people (Elliott, 2019).^2^ Indeed, adolescents and young professionals often search for atypical schedules (desynchronization) in order to resist external constraints and express some originality (Lachance, 2011).

The impression of losing track of time also goes beyond the act of forgetting what day we are. After 6 or 7 weeks of quarantine, it was possible to hear among citizens who had seen their schedule disrupted by the quarantine due to COVID-19, expressing the paradoxical impression that “it has already been x weeks since the beginning of the quarantine,” just as if it went by quickly and, at the same time, got the impression that “it feels as if it started a year ago,”^3^ which was clearly an overestimation of the real duration of the quarantine. This paradox is difficult to understand, but it reveals that there is more than one track in our organization of time in memory. With the amount of new events marking daily life (reports about public health in terms of the number of new cases, serious cases, and deaths in the different regions, provinces, states, or foreign countries; the evolution of recommendations about the tolerated level of quarantine; and the announcements about financial help to different groups…), it is understandable that attention was detracted from time, which theoretically should result in an impression that a given time interval was perceived as short. At the same time, when you look at the duration retrospectively, it is known that the estimated duration seems longer when filled with less routine activities (Avni-Babad and Ritov, 2003). This is also the case when the amount and complexity of information that is being processed during the period whose duration has to be estimated, along with contextual changes, are larger (Block, 1982). In other words, the possibility to access so much information since the beginning of the quarantine contributes to the impression that its beginning is distant (Zauberman et al., 2010).

## Anxiety and Uncertainty

Losing track of time can be charming on vacation, but less so when there is a sword of Damocles over our heads. Two other critical factors also contribute to explaining the temporal confusion people experienced during the quarantine caused by the pandemic of COVID-19. A state of anxiety can disturb the impression of time. When people are placed in conditions where they must wait for an event to occur, which is sufficiently important to increase anxiety, they tend to overestimate their waiting time (Sarason and Stoops, 1978). One interpretation of this effect, in continuity with the pacemaker-counter theory described earlier, is that anxiety likely increased arousal, and arousal is known to accelerate the rate of pulses’ emission by the pacemaker of the internal clock described earlier (Grondin et al., 2014). Such acceleration results in the accumulation of more pulses and, therefore, a longer perceived duration. People who have experienced an increased level of anxiety during the quarantine likely experienced a distortion of their impression of time. There are multiple reasons to believe that one may have had an increased level of anxiety. Being limited in displacements, *per se*, could be stressful, especially if there is not much space at home and many people share this space on a near full-time basis. An even more critical source of anxiety is related to work conditions. Some people have had to try to keep working while having much more to do at home with children. Others were in conditions where they even wondered about the possibility of retrieving the job they lost at the beginning of the quarantine. Not knowing what the future will bring may seriously affect health and well-being (for a brief review, see Holman and Grisham, 2020). Above all of this, some people may have had an increased level of anxiety directly related to the possibility of catching COVID-19 and dying, or because there were cases in the family or at their place of work. This anxiety could also have been generated by the possibility of an elderly parent catching COVID-19 and dying, with the thought of not being able to provide presence and affective support, if not basic care, at this critical moment of existence (Steele, 2020).

The effect of this anxiety factor was magnified by another crucial factor, namely, the uncertainty about the duration of the pandemic. Knowing how much time remains before an endpoint gives us a chance to be prepared at the moment when activities resume. A clear temporal landmark in the future allows the distribution of cognitive and emotional resources over time. Anxiety is actually closely associated with future events, or at least, much more associated with future than with past events (Eysenck et al., 2006). The temporal orientation of people is affected by their level of anxiety (and by their level of depression). The tendency to perceive a temporal distance (a month for instance) in the future as closer than the same distance in the past (Caruso et al., 2013) is clearly exaggerated in people who have anxious personality traits (Rinaldi et al., 2017). Depressed people, on the other hand, are more inclined to turn toward the past than anxious people.

A trick to help understanding the impact of uncertainty on the impression of time is to adopt what we might refer to as the hitchhiker analogy. No hitchhiker who needs to travel a few hundreds of kilometer would complain about having to wait for 20 or 30 min for a ride. The problem of the hitchhiker is that after 19 or 29 min, if no one has yet stopped by, the waiting time remains unpredictable, opening the door for the perspective of a disaster: hours of waiting, and maybe even dusk coming. If a car stops after 21 or even 31 min, the relief will soon lead to the impression that, in retrospect, after all, everything went well, and time has passed rapidly. At the beginning of the quarantine in Canada, people were told that the situation would be reassessed in 2 weeks, and new decisions would eventually be made. However, we were also told that we might need to wait for a cure or a vaccine, before returning to normal life, which would take at least 12 months, if not 2 years, all the while not knowing what the severity and requirements of the quarantine would be over the next weeks and months. This amount of temporal uncertainty sets the table for increasing anxiety and, consequently, disturbed judgments about time.

## Individual Differences

People entered into the quarantine with different perspectives on time, and these individual differences necessarily gave a particular tint to their experience of time during this period (Bisson and Grondin, 2020). For instance, people differ on different features such as punctuality, the inclination to perform several tasks simultaneously (polychronicity), and impatience (Francis-Smythe and Robertson, 1999). While impatience is inversely correlated with agreeableness (Settles et al., 2012), the respect of deadlines is positively related to the conscientiousness dimension of the NEO-FFI (Bisson et al., 2015).

People also differ on another important feature of psychological time called temporal perspective (Zimbardo and Boyd, 1999; Sircova et al., 2014). It is generally reported that individuals have a stronger inclination for one of five dimensions, two being associated with the past, two with the present, and one with the future. The fact that people have different orientations about temporal perspective helps understanding why citizens may have different time experiences during the quarantine after the schedule upheaval. People may have a positive (positive past) or negative (negative past) view of, or attitude toward, the past; may be oriented toward pleasure and the present moment (present hedonistic) or have an attitude of hopelessness in face of life (present fatalistic); or may be more inclined toward future (future-oriented).^4^ Considering that a high score on the “past negative” dimension is closely related to a high score on the anxiety trait (Bisson et al., 2015), it is likely that people entering the quarantine with such a personality perceive time as being long. As well, the “present hedonistic” dimension and a high score relating to the search for sensation or novelty are positively correlated (Boyd and Zimbardo, 2005). Therefore, it is likely that someone scoring high on the “present hedonistic” dimension would have tools for generating activities that would allow them to avoid paying attention to time, and would therefore be in a good position to not see time as long. These people are more likely to finding new activities to fill the void that quarantine may have left in their schedule and are more inclined to have the impression that time periods are brief than people who are more oriented toward the “past negative” dimension. This is even more likely considering that higher scores on the present-hedonistic scale are associated with higher uncertainty scores about time (a lower motivation for giving precise time estimates in an experimental setting; see Grondin et al., 2018). Note that in the context of pandemic, men are reported to be more likely than women to be defined by a time perspective dominated by the “present hedonistic” dimension (Virna and Brahina, 2020).

Finally, it is relevant to remind the fragility of the internal representation of time, even in normal conditions. It is known that people in general are not very good at estimating the time required to do a task. This effect, known as *planning fallacy*, is due to the fact that people do not tend to rely on the duration required to perform similar tasks; they rather emphasize certain aspects of them (Kahneman and Tversky, 1973). People underestimate the duration of tasks to do, especially when these tasks require more than 5 min to complete (Roy et al., 2005), and this is partly caused by the difficulty to consider each sub-component of these tasks (Francis-Smythe, 2006). What is more, motivational factors also contribute to the understanding of planning error (Francis-Smythe and Robertson, 1999). With the disappearance of most of what should be done during the quarantine, even past experiences of time organization become useless, people encounter lack of motivation and are plunged into an impression that the sense of time is blurred.^5^

## Conclusion

Humans from all around the world have been facing a pandemic that has required extreme security measures; people were asked to stay at home and normal life was interrupted. One consequence of the quarantine was that, for a rare moment in their life, many people were facing a blank page and invited to draw a new life. They were given plenty of time for drawing, with several constraints and for an undefined period of time. Untrained to deal with so few temporal landmarks, and having no clear deadlines for retrieving normal life, people suddenly experienced confusion about some aspects of psychological time and fully realized its critical importance in the organization of life. This paper proposes different perspectives which may be used to interpret some of the time-related changes that were experienced in everyday life during quarantine. In this context, the people for whom more free time also meant more boredom may have paid more attention to time. From a pacemaker-counter perspective, time seems to go by more slowly when we are paying more attention to it. Anxiety, as well as the impact that temporal uncertainty about the duration of the quarantine has had on anxiety, is also a factor that may have affected time perception during quarantine. These factors may have altered the rate at which the pacemaker emitted pulses, thereby influencing whether time was perceived as longer or shorter. Moreover, when people make retrospective assessments from past temporal experiences, these judgments are prone to the fallibility of memory processes. This is especially true regarding the conditions surrounding quarantine, where familiar temporal landmarks were unavailable. Finally, quarantine has contributed to the loss of useful cues used for planning. These types of changes can confuse our relation to time, and the magnitude of these disturbances in time perception during quarantine could be interpreted in terms of time-related individual differences.

## Author Contributions

SG proposed the interpretations presented in the manuscript and wrote the initial version of the text. EM-D and P-AR, who are members of an international team in a research project about perception of time in quarantine, discussed the interpretations and contributed to the final version of the text. All authors contributed to the article and approved the submitted version.

### Conflict of Interest

The authors declare that the research was conducted in the absence of any commercial or financial relationships that could be construed as a potential conflict of interest.

## Footnotes

^1^The present paper does not emphasize this passage of time perspective, but it does not mean that the temporal confusion experienced during confinement does not have some roots in this internal life.

^2^This notion is not without reminding the idea of affective attunement, i.e., the tendency to mimic and synchronize each other’s vocalizations, postures, and movements (Fuchs, 2013).

^3^It is not excluded that this paradox depends the individuals’ introspection on their internal life (Droit-Volet and Wearden, 2016).

^4^Zimbardo et al. (2012) propose a sixth dimension, transcendental future, to account for the fact that some people plan their lives with the belief that there is a better life after death.

^5^Other psychological views related to time should be noted. We rarely hear, as much as we have during the quarantine, about the age of people on a daily basis. There were clear indications that the likelihood of fatal consequences of contracting the virus increases with age. Consequently, in some countries, even more restricting rules were imposed specifically to people older than 70, and sometimes even to people older than 65. These age-related restrictions were often repeated, and it was likely difficult for most people not to wonder about their personal age or, for younger people, about the age of their parents or grandparents. The virus reminded us what we already know very well, but succeed to ignore most of the time: we will all die and we are only passing on earth. The virus has relocated within the scope of our consciousness the fact that our own time is limited, is counted. Indeed, the virus interrupted the flow of time, made both the near future and the far future less certain, such temporal disruption or uncertainty having an impact on mental health (for a brief review, see Holman and Grisham, 2020). This focus on time was likely magnified by the references to other pandemics in the history of humanity like the Spanish flu a century ago or cholera almost a century earlier. We are more modern, but still vulnerable, mortal. This internalization of large-scale temporal perspectives, regarding age and history, likely sets the table to make us particularly sensitive to time, and this probably contributed to increase anxiety.
